# What Happens after Inbreeding Avoidance? Inbreeding by Rejected Relatives and the Inclusive Fitness Benefit of Inbreeding Avoidance

**DOI:** 10.1371/journal.pone.0125140

**Published:** 2015-04-24

**Authors:** A. Bradley Duthie, Jane M. Reid

**Affiliations:** Institute of Biological and Environmental Sciences, School of Biological Sciences, University of Aberdeen, Aberdeen, United Kingdom; University of Melbourne, AUSTRALIA

## Abstract

Avoiding inbreeding, and therefore avoiding inbreeding depression in offspring fitness, is widely assumed to be adaptive in systems with biparental reproduction. However, inbreeding can also confer an inclusive fitness benefit stemming from increased relatedness between parents and inbred offspring. Whether or not inbreeding or avoiding inbreeding is adaptive therefore depends on a balance between inbreeding depression and increased parent-offspring relatedness. Existing models of biparental inbreeding predict threshold values of inbreeding depression above which males and females should avoid inbreeding, and predict sexual conflict over inbreeding because these thresholds diverge. However, these models implicitly assume that if a focal individual avoids inbreeding, then both it and its rejected relative will subsequently outbreed. We show that relaxing this assumption of reciprocal outbreeding, and the assumption that focal individuals are themselves outbred, can substantially alter the predicted thresholds for inbreeding avoidance for focal males. Specifically, the magnitude of inbreeding depression below which inbreeding increases a focal male’s inclusive fitness increases with increasing depression in the offspring of a focal female and her alternative mate, and it decreases with increasing relatedness between a focal male and a focal female’s alternative mate, thereby altering the predicted zone of sexual conflict. Furthermore, a focal male’s inclusive fitness gain from avoiding inbreeding is reduced by indirect opportunity costs if his rejected relative breeds with another relative of his. By demonstrating that variation in relatedness and inbreeding can affect intra- and inter-sexual conflict over inbreeding, our models lead to novel predictions for family dynamics. Specifically, parent-offspring conflict over inbreeding might depend on the alternative mates of rejected relatives, and male-male competition over inbreeding might lead to mixed inbreeding strategies. Making testable quantitative predictions regarding inbreeding strategies occurring in nature will therefore require new models that explicitly capture variation in relatedness and inbreeding among interacting population members.

## Introduction

Inbreeding, defined as mating between related individuals, is a pervasive force in evolutionary ecology that is postulated to drive the evolution of mating systems [1–3] and dispersal [4, 5], and to influence population dynamics [6, 7] and the expression and persistence of mutation load [8, 9]. Understanding these phenomena therefore requires a thorough understanding of the evolution and occurrence of inbreeding itself.

Reproducing individuals might exhibit strategies of inbreeding preference or avoidance defined as mating with more or less closely related individuals than expected given random mating, or exhibit inbreeding tolerance defined as random mating with respect to relatedness [10]. In general, the evolution of any such inbreeding strategy is expected to depend on the balance between increased inheritance of identical-by-descent alleles by inbred offspring versus any decrease in survival or reproductive fitness of those inbred offspring due to inbreeding depression. This balance is well understood in the context of the evolution of self-fertilisation versus outcrossing [11–16]. Specifically, in an outcrossing population, a mutant allele causing self-fertilisation (the most extreme degree of inbreeding) is 50% more likely to be inherited identical-by-descent by the selfing individual’s offspring than a homologous wild type allele underlying outcrossing, but resulting inbred offspring will commonly show inbreeding depression [2, 12, 13, 17]. The net inclusive fitness benefit of self-fertilisation, and hence the frequency of the underlying mutant allele, will therefore depend on the balance between increased offspring inheritance of identical-by-descent alleles versus reduced offspring survival or reproductive fitness [12, 13, 15–18].

The net inclusive fitness benefit of biparental inbreeding (i.e., inbreeding between two non-self individuals) depends on this same balance. Inbreeding depression is widespread and can substantially reduce offspring fitness in populations with biparental fertilisation [7, 19, 20]. Consequently, inbreeding depression is widely presumed to drive the evolution of inbreeding avoidance in such populations [12, 21–23]. But the inclusive fitness increment of biparental inbreeding relative to outbreeding—which stems from the higher probability that identical-by-descent alleles will be inherited by inbred offspring—has been less widely factored into verbal or quantitative models regarding the evolution of biparental inbreeding versus inbreeding avoidance [10].

Models examining the evolution of self-fertilisation cannot be directly extrapolated to predict the evolution of biparental inbreeding strategy because they do not account for sex-specific inclusive fitness benefits or differing reproductive strategies between males and females with potentially conflicting evolutionary interests [10]. Instead, Parker [24, 25] provided a basic conceptual model that specifically emphasised the inclusive fitness benefit of biparental inbreeding stemming from increased probability of identity-by-descent, and this model was subsequently extended by Waser et al. [26], Kokko and Ots [27], and Puurtinen [28]. These models emphasise that inbreeding preference or tolerance might be adaptive even when inbreeding depression occurs. They also predict evolutionary sexual conflict over inbreeding, meaning that selection on inbreeding preference, tolerance, or avoidance might differ between males and females [24, 25]. This sexual conflict arises because reproductive investment in inbred offspring differentially affects the mean inclusive fitness of each sex (assuming different sex roles). If an individual of the sex whose reproduction is limited by resource availability inbreeds, resources will be invested in less fit inbred offspring instead of fitter outbred offspring. In contrast, an individual of the sex whose reproduction is limited by mate availability might be able to inbreed without losing outbreeding success, as in systems where the mate-limited sex provides no parental care. Where some care is provided, the relative cost of inbreeding for the mate-limited sex has been modelled and interpreted as an opportunity cost in terms of missed opportunities to outbreed [24, 25]. Because some asymmetric investment in offspring is the norm in systems with biparental reproduction, sexual conflict is predicted to be a fundamental outcome of biparental inbreeding.

However, existing models of biparental inbreeding make strong and restrictive assumptions. Most assume that the focal population primarily comprises outbred and unrelated individuals, but also contains two focal relatives that might or might not inbreed with each other [24–26]. Under these assumptions, if a focal individual avoids inbreeding, then its rejected relative must also avoid inbreeding. These models therefore implicitly assume mutual inbreeding avoidance. In contrast, Puurtinen [28] assumes that females can optimise their inbreeding by selecting males that are related to a specific degree. These conditions are very unlikely to apply in wild populations in which biparental inbreeding might occur. Rather, both non-relatives and relatives of different (but unlikely all optimal) degrees are likely to be available as potential mates. Consequently, relatives that are rejected as mates by a focal individual might subsequently inbreed to varying degrees whether by choice or coercion, or might avoid inbreeding. For example, inbreeding might benefit rejected relatives if the encounter rate between potential mates is low, meaning that avoiding subsequent inbreeding would increase the risk of breeding failure [27, 29]. Alternatively, rejected females might be subsequently coerced into mating with another relative [24, 25, 30]. Therefore, a focal individual that is ‘deciding’ whether or not to inbreed cannot necessarily ensure or assume that its rejected relative will subsequently outbreed.

The degree to which rejected relatives subsequently inbreed is likely to be critical to inclusive fitness calculations. Conceptual models that assume reciprocal inbreeding avoidance [24–26] or the availability of optimally related kin [28] would have limited applicability or predictive ability when the inclusive fitness benefits of inbreeding versus avoiding inbreeding are conditional upon the subsequent mating decisions of rejected relatives. Nevertheless, multiple studies have attempted to apply quantitative predictions derived from existing models of biparental inbreeding [24–27] to empirical systems [31–34]. Here we show that these predictions are unlikely to be accurate when highly restrictive assumptions are violated.

We first formally summarise and review Parker’s [24, 25] basic conceptual model (hereafter ‘Parker’s model’) that illustrates the balance between the inclusive fitness benefit of biparental inbreeding and inbreeding depression. We then extend this model in three ways. First, we relax the assumption that focal individuals are outbred. Second, we relax the assumption that the rejected relatives of focal individuals will subsequently outbreed. Finally, we demonstrate that indirect opportunity costs may exist if a focal male’s rejected potential mate subsequently breeds with his male relative. We discuss how our model relates to empirical and theoretical research on biparental inbreeding, and to the broader context of inclusive fitness theory. We thereby highlight new avenues of research for biparental inbreeding theory, including understanding family dynamics and ultimately a more predictive evolutionary theory of inbreeding strategy.

## Parker’s model

Parker’s model evaluates the (inclusive) fitness costs and benefits of the decision to inbreed or avoid inbreeding for each sex. For convenience, the sexes whose reproduction is limited by mate availability and resource availability are labelled as ‘male’ and ‘female’, respectively. In the most basic model, males can mate with any number of females during a reproductive bout to increase their reproductive success, but females can only mate with one male and produce *n* offspring, which is assumed constant for all females and reproductive bouts. Mates can be unrelated and produce outbred offspring, or be related by some degree and produce correspondingly inbred offspring.

**Fig 1:**
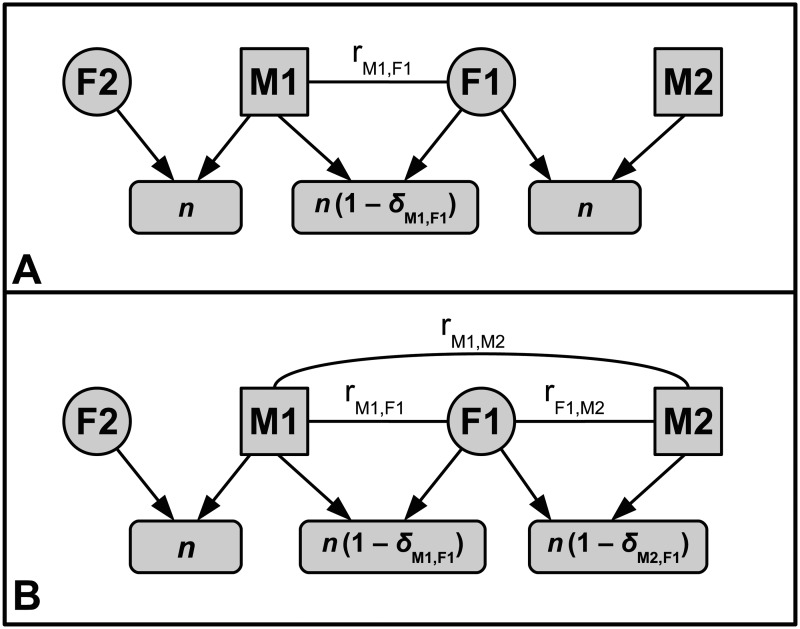
Conceptual models of biparental inbreeding. Females (*F1* and *F2*) produce *n* offspring. The focal male (*M1*) is related by *r_M1,F1_* to the focal female (*F1*). The fitness of *M1* and *F1*’s *n* offspring is decreased by inbreeding depression *δ*_*M*1,*F*1_. Whether or not *M1* inbreeds or avoids inbreeding with *F1* does not affect his opportunity to outbreed with an unrelated female (*F2*). In Parker’s [24, 25] model (A), if *M1* avoids inbreeding, then *F1* is assumed to outbreed with unrelated male (*M2*). In our extended model (B), if *M1* avoids inbreeding, *F1*’s alternative mate *M2* may be related to *M1* (*r_M1,M2_*) or *F1* (*r_M2,F1_*). If *r*_*M*2,*F*1_ > 0, then the fitness of *M2* and *F1*’s offspring will be decreased by inbreeding depression (*δ*_*M*2,*F*1_).

Parker’s model considers a potentially inbreeding focal male (*M1*) and focal female (*F1*) that are related by some degree *r_M1,F1_* (Fig 1A). All other potential mates of *M1* and *F1* are assumed to be unrelated to both *M1* and *F1*. Following inbreeding avoidance by one focal individual (*M1* or *F1*), the other focal individual is therefore assumed to outbreed [24–26]. For example, if *M1* avoids inbreeding with *F1*, then *F1* is assumed to outbreed with an alternative male *M2* (Fig 1A). The most basic model assumes that *M1*’s opportunity to outbreed with unrelated females (e.g., *F2* in Fig 1A), is unaffected by whether or not he inbreeds or avoids inbreeding with *F1*, so additional offspring that *M1* might sire do not need to be included in inclusive fitness calculations. In contrast, if *F1* inbreeds with *M1*, she cannot also outbreed.

Parker [24, 25] calculated the inclusive fitness consequences of inbreeding versus avoiding inbreeding for *M1* and *F1*. When *M1* avoids inbreeding with *F1*, a proportion *r_M1,F1_* of *F1*’s direct allelic contribution to her offspring will be identical-by-descent with alleles carried by *M1*. *M1*’s inclusive fitness increment from avoiding inbreeding is therefore $\frac{n}{2}\times r_{M1,F1}$. Alternatively, if *M1* inbreeds with *F1*, identical-by-descent alleles will be contributed to inbred offspring by both *M1* ($\frac{n}{2}$) and *F1* ($\frac{n}{2}\times r_{M1,F1}$). This increases *M1*’s inclusive fitness increment to $\frac{n}{2}+\frac{n}{2}\times r_{M1,F1}$. But this inclusive fitness increment due to inbreeding is decreased by inbreeding depression such that inbred offspring produced by *M1* and *F1* have (1 − *δ*_*M*1,*F*1_) the fitness of outbred offspring [24–27]. The magnitude of inbreeding depression below which inbreeding benefits *M1* is given by the values of *δ*_*M*1,*F*1_ for which the inclusive fitness gain from inbreeding exceeds that of avoiding inbreeding [24–26],

$$\begin{array}{c} \underset{\begin{array}{c} \text{Fitness}\;\text{increment}\\ \text{when}\;\text{M1}\;\text{inbreeds}\\ \text{with}\;\text{F1} \end{array}}{\underset{︸}{\frac{n}{2}(1+r_{M1,F1})}}\underset{\begin{array}{c} \text{Reduced}\;\text{fitness}\\ \text{of}\;\text{M1}\;\&\;\text{F1}’\text{s}\\ \text{offspring} \end{array}}{\underset{︸}{\left(1-\delta_{M1,F1}\right)}}>\underset{\begin{array}{c} \text{Fitness}\;\text{if}\\ \text{M1}\;\text{avoids}\\ \text{inbreeding} \end{array}}{\underset{︸}{\frac{n}{2}(r_{M1,F1})}}. \end{array}$$ (1)

**Fig 2:**
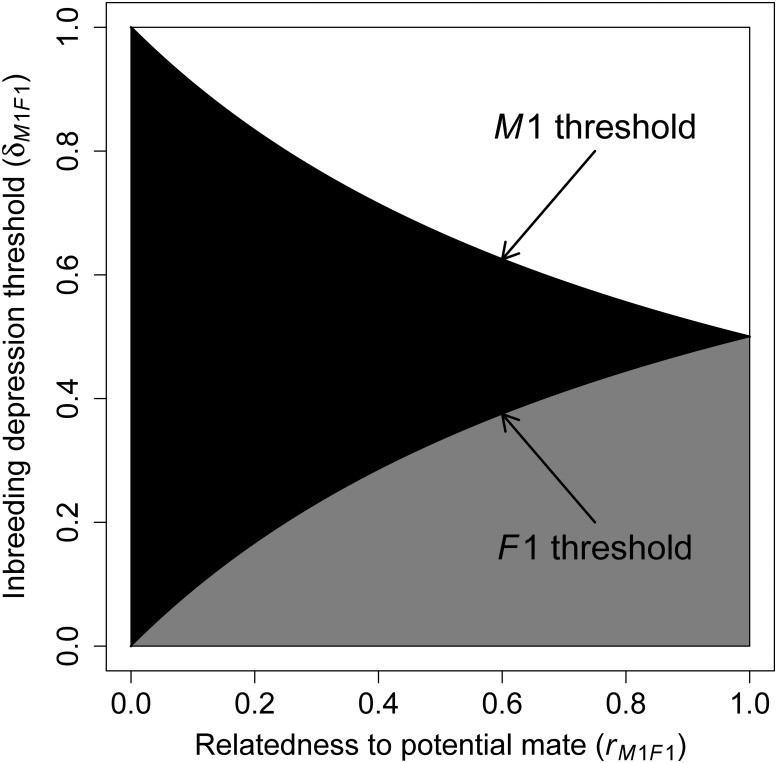
Zones of parameter space in which inbreeding versus avoiding inbreeding is predicted to increase male and female inclusive fitness when all other potential mates are unrelated. The x-axis shows the relatedness (*r*_*M*1,*F*1_) between two focal potential mates, male *M1* and female *F1*, where *r*_*M*1,*F*1_ = 0 equates to outbreeding, and *r*_*M*1,*F*1_ = 1 equates to self-fertilisation. The y-axis shows the magnitude of inbreeding depression (*δ*_*M*1,*F*1_) below which inbreeding is beneficial for each sex. Areas where neither sex, both sexes, and males only benefit from inbreeding are shown in white, grey, and black, respectively. The intersections between black and white areas and black and grey areas respectively demarcate the thresholds below which *M1* and *F1* benefit by inbreeding [24, 25].

Fig 2 shows threshold values of *δ*_*M*1,*F*1_ below which inbreeding increases *M1*’s inclusive fitness given different values of *r_M1,F1_*. For the widely cited illustrative example where $r_{M1,F1}=\frac{1}{2}$ (e.g., *M1* and *F1* are full siblings with unrelated parents), *M1*’s inclusive fitness from inbreeding exceeds that from avoiding inbreeding if $\delta_{M1,F1}<\frac{2}{3}$ [10, 24–27].

From the perspective of *F1*, the inclusive fitness gain from inbreeding with *M1* versus avoiding inbreeding can be calculated similarly [24, 25]. Because *M1*’s additional outbreeding success is assumed to be unaffected by inbreeding with *F1*, it is irrelevant for calculating *F1*’s inclusive fitness. If *F1* avoids inbreeding with *M1* and outbreeds with *M2* instead, her inclusive fitness is $\frac{n}{2}$ (Fig 1A). If *F1* inbreeds with *M1*, her inclusive fitness is $\frac{n}{2}\left(1+r_{M1,F1}\right)(1-\delta_{M1,F1})$. Inbreeding with *M1* is therefore beneficial for *F1* if,

$$\begin{array}{c} \frac{n}{2}(1+r_{M1,F1})\left(1-\delta_{M1,F1}\right)>\frac{n}{2}. \end{array}$$ (2)

Fig 2 shows that the threshold of inbreeding depression below which inbreeding benefits *F1* is symmetrical to that for *M1*. For example, when *F1* and *M1* are full siblings, inbreeding benefits *F1* if $\delta_{M1,F1}<\frac{1}{3}$, compared to $\frac{2}{3}$ for *M1* [10, 24–27].

Parker’s model could therefore be interpreted to predict that inbreeding tolerance or preference can be adaptive for one or both sexes, even given non-zero inbreeding depression (*δ*_*M*1,*F*1_ > 0; Fig 2). It also predicts a zone of sexual conflict, where inbreeding increases male fitness but decreases female fitness for a substantial range of *δ*_*M*1,*F*1_ (black zone, Fig 2). This zone is greatest given small *r_M1,F1_* values and decreases to zero as *r_M1,F1_* approaches 1 (self-fertilisation).

## Relaxing the assumption that focal individuals are outbred

Parker’s model highlights that *r_M1,F1_* is a key parameter underlying inclusive fitness calculations for inbreeding versus avoiding inbreeding, but *r_M1,F1_* is rarely explicitly defined in biparental inbreeding theory (but see [10]). Parker’s [24] original derivation does not include *r_M1,F1_* at all, and focuses only on the specific case in which potential mates share exactly half their alleles (e.g., outbred full siblings). Waser et al. [26] explicitly equated *r_M1,F1_* to Wright’s [35] coefficient of relationship, while Parker [25] and Kokko and Ots [27] define *r_M1,F1_* as ‘relatedness’ without further elaboration. Clearly, in order to calculate appropriate thresholds for inbreeding avoidance or preference (Ineqs. 1 and 2), relatedness must be appropriately defined for inclusive fitness calculations.

In particular, it is not always appreciated that the relevant *r_M1,F1_* values depend on the degree to which focal individuals are themselves inbred. The alternative implicit assumption, that all focal individuals are outbred, is unlikely to be valid in populations where biparental inbreeding might occur. The inclusive fitness consequences of inbreeding versus avoiding inbreeding therefore need to be considered for focal individuals that are themselves inbred to some degree, by defining *r_M1,F1_* appropriately. In their developments of Parker’s model, Lehmann and Perrin [36] and Puurtinen [28] account for inbred focal individuals by defining *r_M1,F1_* as the regression coefficient of relatedness [37, 38]. But, to our knowledge, this definition has not been explicitly applied to Parker’s basic model.

If focal individuals are themselves inbred, then two homologous alleles have a non-zero probability of occurring identical-by-descent within an individual. Specifically, the probability that two of *M1*’s homologous alleles are identical-by-descent defines his coefficient of inbreeding *f_M1_*. The probability that an allele is directly transmitted to *M1*’s offspring then increases from $\frac{1}{2}$ to $\frac{1}{2}\left(1+f_{M1}\right)$. The regression coefficient of relatedness (henceforth denoted *r_M1,F1_*) is the proportion of *M1*’s alleles expected to be carried identical-by-descent by *F1* [38]. Therefore, *M1*’s relatedness to *F1* is,

$$\begin{array}{c} r_{M1,F1}=\frac{2f_{M1,F1}}{1+f_{M1}}. \end{array}$$ (3)

The coefficient of kinship *f_M1,F1_* (sometimes denoted as *k_M1,F1_* or *θ* *_M1,F1_*) is the probability that an allele randomly sampled from *M1* is identical-by-descent with a homologous allele randomly sampled from *F1*, and is therefore symmetrical between two individuals (i.e., *f*_*M*1,*F*1_ = *f*_*F*1,*M*1_). But if two individuals *M1* and *F1* are inbred to different degrees (*f*_*M*1_ ≠ *f*_*F*1_), their relatedness—calculated in the context of inclusive fitness [37]—may be asymmetrical (*r*_*M*1,*F*1_ ≠ *r*_*F*1,*M*1_), so that *F1*’s relatedness to *M1* is,

$$\begin{array}{c} r_{F1,M1}=\frac{2f_{F1,M1}}{1+f_{F1}}. \end{array}$$ (4)

This asymmetry occurs because the denominators of Eqs (3) and (4) contain *M1* and *F1*’s coefficients of inbreeding, respectively, which will differ if *M1* and *F1* are inbred to different degrees.

We now reformulate Parker’s model using Eqs (3) and (4) to show how *f_M1,F1_*, *f_M1_*, and *f_F1_* affect the predicted thresholds for inbreeding avoidance versus tolerance or preference. Substituting Eq (3) into Ineq. (1) for *M1* yields the inbreeding depression threshold below which *M1* benefits by inbreeding with *F1*,

$$\begin{array}{c} \delta_{M1,F1}<\frac{1+f_{M1}}{1+f_{M1}+2f_{M1,F1}}. \end{array}$$ (5)

Substituting Eq (4) into Ineq. (2) gives the inbreeding depression threshold below which *F1* benefits by inbreeding with *M1*,

$$\begin{array}{c} \delta_{M1,F1}<\frac{2f_{F1,M1}}{1+f_{F1}+2f_{F1,M1}}. \end{array}$$ (6)

These expressions show that the thresholds for inbreeding versus inbreeding avoidance depend directly on the coefficient of inbreeding of each focal individual, but do not depend on that of their potential mate. Increasing *f_M1_* increases the right hand side of Ineq. (5), whereas increasing *f_F1_* decreases the right hand side of Ineq. (6). Inbred males therefore benefit by inbreeding at higher thresholds of *δ*_*M*1,*F*1_ than outbred males, and inbred females benefit by avoiding inbreeding at higher *δ*_*M*1,*F*1_ thresholds than outbred females. Our extension of Parker’s model therefore shows that the degree to which focal individuals are inbred cannot be ignored when calculating relatedness, and hence when inferring thresholds defining sex-specific inbreeding strategy.

## Relaxing the assumption of mutual inbreeding avoidance

While Parker’s model illustrates that inbreeding can be adaptive and that sexual conflict over inbreeding might occur, any quantitative conclusions are limited by the strong assumption that rejected relatives will not inbreed. We now extend Parker’s model to show that subsequent inbreeding by a rejected relative can greatly alter the inclusive fitness benefit of avoiding inbreeding for a focal male, and therefore alter the benefit of inbreeding versus inbreeding avoidance and change the expected magnitude of sexual conflict over inbreeding.

To relax the assumption that the focal *M1*’s rejected relative *F1* will outbreed, we allow *F1*’s alternative mate *M2* to be related to *M1*, *F1*, or both by *r_M1,M2_* and *r_F1,M2_*, respectively (Fig 1B). We limit our model to one additional relative (*M2*), rather than many differently related individuals for tractability. Our current aim is simply to demonstrate that an additional relative can affect inbreeding depression thresholds defining inbreeding strategies, not to predict evolutionary outcomes that might arise given realistic relatedness distributions within populations; we therefore do not assume that a focal population comprises only three relatives, or that this situation will persist over evolutionary time (see Discussion).

If *F1* breeds with *M2* after *M1* avoids inbreeding with her, then inbreeding with *F1* is beneficial for *M1* if,

$$\begin{array}{c} \underset{\begin{array}{c} \text{Fitness}\;\text{increment}\\ \text{when}\;\text{M1}\\ \text{inbreeds} \end{array}}{\underset{︸}{\frac{n}{2}(1+r_{M1,F1})}}\underset{\begin{array}{c} \text{Reduced}\;\text{fitness}\\ \text{of}\;\text{M1}\;\&\;\text{F1}’\text{s}\\ \text{offspring} \end{array}}{\underset{︸}{\left(1-\delta_{M1,F1}\right)}}>\underset{\begin{array}{c} \text{Fitness}\;\text{increment}\\ \text{when}\;\text{M1}\;\text{avoids}\\ \text{inbreeding}\;\text{with}\;\text{F1} \end{array}}{\underset{︸}{\frac{n}{2}(r_{M1,F1}+r_{M1,M2})}}\underset{\begin{array}{c} \text{Reduced}\;\text{fitness}\\ \text{of}\;\text{M2}\;\&\;\text{F1}’\text{s}\\ \text{offspring} \end{array}}{\underset{︸}{\left(1-\delta_{M2,F1}\right)}}. \end{array}$$ (7)

In Ineq. (7), *δ*_*M*2,*F*1_ is the inbreeding depression in offspring produced by *M2* and *F1*. The right hand side of Ineq. (7) reduces to that of Ineq. (1) when *M2* is unrelated to *M1* and *F1* (i.e., *r*_*M*1,*M*2_ = 0 and *δ*_*M*2,*F*1_ = 0). The inclusive fitness gain *M1* receives by inbreeding with *F1* remains the same as in Parker’s model (left hand side of Ineqs. 1 and 7) because *r_M1,F1_* and *δ*_*M*1,*F*1_ are unchanged (Fig 1A and 1B). Inequality (Eq 7) shows that *M1*’s fitness gain from avoiding inbreeding with *F1* changes if *F1* subsequently breeds with a related *M2* (whether by choice or coercion). This is because *r_M1,M2_* or *δ*_*M*2,*F*1_, or both, will be non-zero and hence affect *M1*’s inclusive fitness. Although *F1* will pass alleles to her offspring produced with *M2* that are identical-by-descent to alleles carried by *M1*, the indirect fitness benefit to *M1* will be decreased by any inbreeding depression in *F1*’s offspring that occurs when *F1* inbreeds with a related *M2*. But if *M1* avoids inbreeding with *F1*, he can potentially gain indirect fitness through *F1*’s alternative mate *M2* if *M1* and *M2* are related (i.e., *r*_*M*1,*M*2_ > 0; Fig 1B). Explicitly considering non-zero relatedness among *M1*, *F1*, and *M2* can therefore increase or decrease *M1*’s inclusive fitness gain from inbreeding versus avoiding inbreeding.

In contrast, alternative female mates of *M1* (and *M2*) do not need to be considered when determining whether *F1*’s inclusive fitness is increased by inbreeding with *M1* versus *M2* if the outbreeding success of both males is unaffected by *F1*’s decision. The conditions where inbreeding with *M1* increases *F1*’s inclusive fitness more than breeding with *M2* can be found by comparing *F1*’s relatedness to each male and the inbreeding depression in the resulting offspring. Inbreeding with *M1* instead of *M2* increases the inclusive fitness of *F1* if,

$$\begin{array}{c} \frac{n}{2}(1+r_{F1,M1})\left(1-\delta_{M1,F1}\right)>\frac{n}{2}(1+r_{F1,M2})\left(1-\delta_{M2,F1}\right). \end{array}$$ (8)

The left hand side of Ineq. (8) is identical to that of Ineq. (2), and the right hand side reduces to that of Ineq. (2) when *r*_*F*1,*M*2_ = 0 and *δ*_*M*2,*F*1_ = 0.

To illustrate the inclusive fitness consequences that result from relaxing Parker’s [24, 25] assumption of reciprocal inbreeding avoidance, we first consider three specific scenarios. We use these scenarios as examples to illustrate why the existence of additional related potential mates is important for predicting the evolution of inbreeding strategy, not to make specific quantitative predictions for any particular species. Furthermore, the three focal individuals considered in each scenario are not assumed to be the only individuals within a focal population, nor are their relatedness combinations assumed to be representative of the full relatedness structure of a larger population. Our objective here is simply to show that the presence of a related potential mate affects inclusive fitness calculations. Scenario 1 illustrates a case in which all three focal individuals are equally related. Scenario 2 introduces an example in which relatedness differs among all three focal individuals. Scenario 3 illustrates that when *F1* is related to *M1* and *M2*, both *δ*_*M*1,*F*1_ and *δ*_*M*2,*F*1_ affect inbreeding depression thresholds. Following the three illustrative scenarios, we define inbreeding depression as a function of kinship to facilitate comparison with Parker’s model, and provide a systematic summary of the implications of Ineqs. (7) and (8). For simplicity, our illustrative scenarios assume that focal individuals are outbred, but this is not a condition of Ineqs. (7) and (8) given that relatedness is appropriately defined.

### Scenario 1: M1, F1, and M2 are all equally related

**Fig 3:**
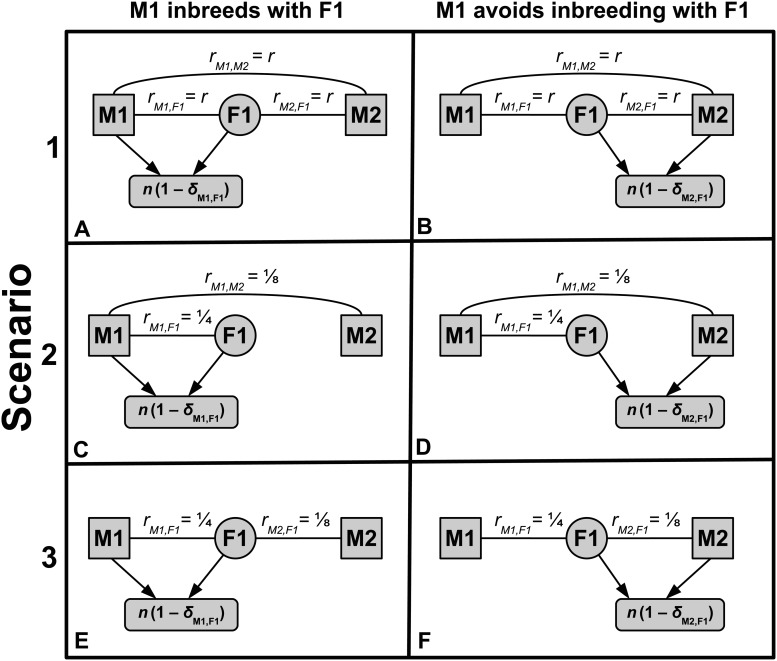
Three illustrative scenarios of biparental inbreeding in which the inclusive fitness benefits to a focal male *M1* and female *F1* depend on their relatedness to the female’s alternative mate *M2*. *F1* produces *n* offspring, whose fitness is reduced by inbreeding depression *δ*_*M*1,*F*1_ or *δ*_*M*2,*F*1_ if she mates with *M1* or *M2*, respectively. In scenario 1 (A and B), *M1*, *F1*, and *M2* are all equally related such that *r*_*M*1,*F*1_ = *r*_*M*1,*M*2_ = *r*_*M*2,*F*1_ = *r*. In scenario 2 (C and D), *M1* and *F1* are half-siblings, *M1* and *M2* are first cousins, and *M2* and *F1* are unrelated. In scenario 3 (E and F), *M1* and *F1* are half-siblings, *M1* and *F1* are first cousins, and *M1* and *M2* are unrelated.

If *M1*, *F1*, and *M2* are all equally related (Fig 3A and 3B), then the magnitude of inbreeding depression in *F1*’s offspring can be assumed to be the same whether she mates with *M1* or *M2* (*δ*_*M*1,*F*1_ = *δ*_*M*2,*F*1_). Solving Ineq. (7) given these conditions reveals that inbreeding with *F1* increases *M1*’s inclusive fitness more than avoiding inbreeding with *F1* if *δ*_*M*1,*F*1_ < 1. Therefore, assuming there are no additional costs of inbreeding, *M1* will never benefit from avoiding inbreeding if *F1* subsequently inbreeds with an equally close relative of both *M1* and *F1*. This conclusion contrasts with Parker’s model that assumes *M2* is unrelated to *M1* and *F1* (Fig 1A), and which predicts that *M1*’s inclusive fitness is increased by avoiding inbreeding given sufficiently high *δ*_*M*1,*F*1_ (Fig 2).

From *F1*’s point of view, there is no fitness difference between inbreeding with *M1* versus *M2* because *r*_*F*1,*M*1_ = *r*_*F*1,*M*2_ and hence *δ*_*M*1,*F*1_ = *δ*_*M*2,*F*1_ (Ineq. 8; Fig 1A and 1B). If an unrelated *M*3 is also available, then the threshold of inbreeding depression below which inbreeding with *M1* or *M2* is beneficial for *F1* does not change from Parker’s model (Ineq. 2; Fig 2).

### Scenario 2: M1 and F1 are half-siblings; M1 and M2 are cousins

In the scenario illustrated in Fig 3C and 3D, *M1* and *F1* are outbred half-siblings ($r_{M1,F1}=\frac{1}{4}$), *M1* and *M2* are outbred first cousins ($r_{M1,M2}=\frac{1}{8}$), and *M2* and *F1* are unrelated (*r*_*M*2,*F*1_ = 0). Because *M2* and *F1* are unrelated, there is no inbreeding depression in *M2* and *F1*’s offspring (*δ*_*M*2,*F*1_ = 0). Solving Ineq. (7) for *δ*_*M*1,*F*1_ shows that *M1* increases his inclusive fitness more by inbreeding with *F1* rather than avoiding inbreeding with her if $\delta_{M1,F1}<\frac{7}{10}$. This threshold is lower than that predicted by Parker’s model ($\delta_{M1,F1}<\frac{4}{5}$), which assumes that *M2* is unrelated to *M1* and *F1* (Fig 2). The two thresholds differ because *r*_*M*1,*M*2_ > 0. This scenario illustrates that when *M1* and *M2* are related, but *F1* and *M2* are not, the threshold value of inbreeding depression below which inbreeding with *F1* benefits *M1* is lower than predicted by Parker’s model. In contrast, because *F1* and *M2* are unrelated, *F1* increases her inclusive fitness by inbreeding with *M1* at the same values of *δ*_*M*1,*F*1_ as predicted by Parker’s model (Fig 1A; Ineq. 2), namely $\delta_{M1,F1}<\frac{1}{5}$. The combined decrease in *M1*’s inbreeding depression threshold and lack of change in *F1*’s threshold decreases the zone of *δ*_*M*1,*F*1_ values over which sexual conflict exists compared to Parker’s model.

### Scenario 3: M1 and F1 are half siblings: M2 and F1 are cousins

In a final illustrative scenario, *M1* and *F1* are outbred half-siblings, and *M2* is an outbred cousin of *F1* and unrelated to *M1*, such that $r_{M1,F1}=\frac{1}{4}$, *r*_*M*1,*M*2_ = 0, and $r_{F1,M2}=\frac{1}{8}$ (Fig 3E and 3F). Here *F1*’s offspring will be inbred whether she mates with *M1* or *M2*. Therefore, it is likely that *δ*_*M*1,*F*1_ > 0 and *δ*_*M*2,*F*1_ > 0, so both will affect the conditions under which *M1* increases his inclusive fitness by inbreeding versus avoiding inbreeding with *F1* (Fig 3E and 3F). From Ineq. (7), *M1* increases his inclusive fitness by inbreeding with *F1* if 4 > 5*δ*_*M*1,*F*1_ − *δ*_*M*2,*F*1_. In contrast, *F1* will benefit by inbreeding with her half-brother *M1* rather than her cousin *M2* if 1 > 10*δ*_*M*1,*F*1_ − 9*δ*_*M*2,*F*1_ (Ineq. 8). Any general solution to these inequalities requires a comparison of *δ*_*M*1,*F*1_ and *δ*_*M*2,*F*1_, which requires a function relating parental kinship to the magnitude of inbreeding depression in offspring. We now provide such a function, and thereby provide a systematic analysis of Ineqs. (7) and (8).

### Systematic analysis of inbreeding avoidance thresholds

To better illustrate inbreeding depression thresholds and facilitate comparison with Parker’s model for *M1* and *F1* when *M2* may be related to either or both, we define inbreeding depression as a function of *M1* and *F1*’s coefficient of kinship *f_M1,F1_*, which is equal to their offsprings’ coefficient of inbreeding (*f_off_*). From first principles, offspring fitness (*W_off_*) is expected to decrease as a log-linear function of *f_off_* such that ln(*W*_*off*_) = −*β*_0_ − *β*_1_ *f*_*off*_, assuming that genetic and environmental influences on fitness are independent and that genetic effects are multiplicative across loci [7, 39, 40]. The slope *β*_1_ measures the load expressed due to inbreeding, and the intercept *β*_0_ can be interpreted as a measure of load that is independent of inbreeding, but potentially includes environmental and genetic effects expressed in an outbred population [41]. The fitness of offspring produced by *M1* and *F1* can therefore be defined as,

$$\begin{array}{c} e^{-(\beta_{0}+\beta_{1}f_{M1,F1})}=\left(1-\delta_{M1,F1}\right). \end{array}$$ (9)

By substituting Eq (9) into Ineq. (7) and solving for *β*_1_, the conditions under which *M1* increases his inclusive fitness by inbreeding versus avoiding inbreeding with *F1* can be derived in terms of coefficients of kinship and inbreeding instead of *δ*_*M*1,*F*1_ and *δ*_*M*2,*F*1_,

$$\begin{array}{c} \beta_{1}^{M1}<\frac{-\ln[\frac{2(f_{M1,F1}+f_{M1,M2})}{\left(1+2f_{M1,F1}+f_{M1}\right)}]}{f_{M1,F1}-f_{M2,F1}}. \end{array}$$ (10)

The analogous condition under which a focal female *F1* increases her inclusive fitness by inbreeding versus avoiding inbreeding with *M1* is given by substituting Eq (9) into Ineq. (8),

$$\begin{array}{c} \beta_{1}^{F1}<\frac{-\ln[\frac{1+f_{F1}+2f_{M2,F1}}{1+f_{F1}+2f_{M1,F1}}]}{f_{M1,F1}-f_{M2,F1}}. \end{array}$$ (11)

Inequalities Eqs (10) and (11) define threshold inbreeding depression slopes below which inbreeding is beneficial for *M1* and *F1*, respectively, given that *F1*’s alternative mate *M2* might be related to *M1*, *F1*, or both. These inequalities can be used to compare threshold values that previously could only be expressed in terms of both *δ*_*M*1,*F*1_ and *δ*_*M*2,*F*1_. For example, given scenario 3 (Fig 3E and 3F), inbreeding with *F1* increases *M1*’s inclusive fitness more than avoiding inbreeding with her if $\beta_{1}^{M1}<25.75$. By contrast, Parker’s model predicts $\beta_{1}^{M1}<12.88$ assuming $f_{M1,F1}=\frac{1}{8}$, *f*_*M*1,*M*2_ = 0, and *f*_*M*2,*F*1_ = 0. From the perspective of *F1*, inbreeding with *M1* increases *F1*’s inclusive fitness more than inbreeding with *M2* if $\beta_{1}^{F1}<1.69$ given scenario 3, but Parker’s model predicts $\beta_{1}^{F1}<1.79$. Our example scenario 3 therefore illustrates that the presence of a related *M2* can affect the threshold slopes below which inbreeding is beneficial for a focal *M1* and *F1*, potentially increasing the predicted zone of sexual conflict over inbreeding.

**Fig 4:**
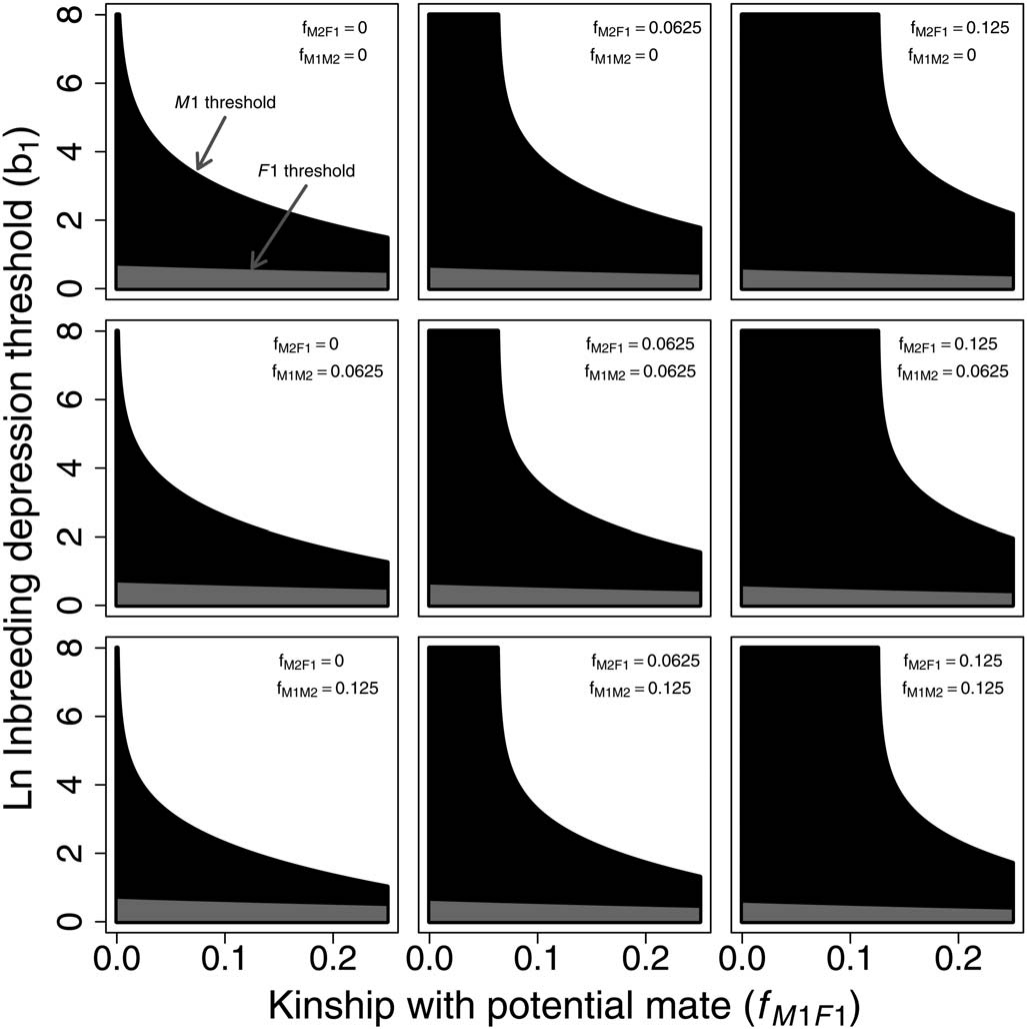
Zones of parameter space in which inbreeding versus inbreeding avoidance is predicted to increase male and female inclusive fitness given varying kinship between *M1*, *F1*, and *M2*. Inbreeding depression thresholds (y-axis shown on a natural log scale) illustrate the values below which *M1* and *F1* have higher inclusive fitness by inbreeding instead of avoiding inbreeding. If *M1* and *F1* do not inbreed, *F1* is assumed to breed with *M2*, who may or not be related to *M1* or *F1*. The kinship between *M1* and *F1* (*f_M1,F1_*) increases along the x-axis of all plots. *f_M2,F1_* and *f_M1,M2_* increase through 0, 0.0625, and 0.125 across left to right columns and top to bottom rows, respectively. Areas where neither sex, both sexes, and males only benefit from inbreeding are shown in white, grey, and black, respectively. Negative threshold values are mathematically possible for some parameter combinations, but are biologically unrealistic because they require that offspring fitness increases monotonically with inbreeding. Where negative thresholds would be required, inbreeding is therefore assumed to never be beneficial. For simplicity, these examples assume focal individuals are outbred.

Moving away from the three simple illustrative scenarios, a more systematic analysis of inbreeding depression thresholds can be obtained using Ineqs. (10) and (11) for different kinship and inbreeding coefficients among *M1*, *F1*, and *M2*. Fig 4 illustrates how $\beta_{1}^{M1}$ and $\beta_{1}^{F1}$ vary with *f_M1,F1_*, *f_M1,M2_*, and *f_M2,F1_*. For ease of illustration, these figures assume that focal individuals are outbred (*f*_*M*1_ = 0 and *f*_*F*1_ = 0), but Ineqs. (10) and (11) do not require these conditions. Each panel shows a single constant combination of *f_M2,F1_* and *f_M1,M2_* values. The top left panel is equivalent to the classic figure derived from Parker’s model (i.e., Fig 2) from *r*_*M*1,*F*1_ = 0 to *r*_*M*1,*F*1_ = 0.5 on the x-axis, but with sex-specific thresholds for inbreeding versus inbreeding avoidance and resulting sexual conflict expressed in terms of ln(*β*_1_) rather than *δ*_*M*1,*F*1_. Fig 4 shows that *f_M2,F1_* and *f_M1,M2_* both affect the conditions under which inbreeding with *F1* benefits *M1* (Ineq. 10). Increases in *f_M2,F1_* (columns from left to right) increase $\beta_{1}^{M1}$, while increases in *f_M1,M2_* (rows from top to bottom) decrease $\beta_{1}^{M1}$. In contrast, increases in *f_M2,F1_* (columns from left to right) decrease $\beta_{1}^{F1}$, but *f_M1,M2_* does not affect $\beta_{1}^{F1}$ (Ineq. 11). Fig 4 also shows that the zone of sexual conflict over inbreeding between *M1* and *F1* (black zone) increases with increasing *f_M2,F1_* (left to right) due to higher inbreeding depression in *F1*’s offspring when *F1* mates with her relative *M2* (which reduces *M1*’s inclusive fitness).

Overall, Fig 4 shows that relaxing one highly restrictive assumption of Parker’s model, that *f*_*M*2,*F*1_ = 0 and *f*_*M*1,*M*2_ = 0, can alter male and female thresholds of inbreeding avoidance and hence the predicted zone of sexual conflict. Although Fig 4 assumes that focal individuals are outbred, inspection of Ineqs. (10) and (11) shows that increasing *f_M1_* increases the threshold below which *M1* benefits by inbreeding with *F1*, whereas increasing *f_F1_* decreases the threshold below which *F1* benefits by inbreeding with *M1*. These patterns remain similar when a linear (i.e., *W* = −*β*_0_ − *β*_1_ *f*_*off*_) rather than a log-linear relationship between parental kinship and offspring fitness is assumed (Supporting Information S1 File). The effects of coefficients of kinship and inbreeding on inbreeding depression thresholds are therefore likely to be robust to different functions relating inbreeding to offspring fitness.

## Opportunity cost of male inbreeding

Thus far, Parker’s basic model and our extensions to consider inbred focal individuals and related alternative males assumed that males do not forgo any additional outbreeding opportunities by inbreeding. This assumption may be unrealistic; if *M1* inbreeds with *F1*, his opportunity to mate with *F2* might decrease (Fig 1A). Parker [24, 25] showed that *M1*’s inclusive fitness gain through siring *F2*’s offspring will then decrease by some fraction, *c*, such that the cost of inbreeding to *M1* is $\frac{nc}{2}$ (*c* can also be interpreted as approximately the ratio of male to female parental investment [24]). If *c* = 1, *M1*’s fitness from outbreeding is completely lost if he inbreeds with *F1* because both sexes invest equally and exclusively to produce *n* offspring. This cost of inbreeding for *M1* can be included in Parker’s model [24–26] so that,

$$\begin{array}{c} \underset{\begin{array}{c} \text{Fitness}\;\text{increment}\\ \text{when}\;\text{M1}\\ \text{inbreeds} \end{array}}{\underset{︸}{\frac{n}{2}(1+r_{M1,F1})}}\underset{\begin{array}{c} \text{Reduced}\;\text{fitness}\\ \text{of}\;\text{M1}\;\&\;\text{F1}’\text{s}\\ \text{offspring} \end{array}}{\underset{︸}{\left(1-\delta_{M1,F1}\right)}}-\underset{\begin{array}{c} \text{M1}’\text{s}\\ \text{mating}\\ \text{cost} \end{array}}{\underset{︸}{\frac{nc}{2}}}>\underset{\begin{array}{c} \text{Fitness}\;\text{increment}\\ \text{when}\;\text{M1}\\ \text{avoids}\;\text{inbreeding} \end{array}}{\underset{︸}{\frac{n}{2}(r_{M1,F1})}}. \end{array}$$ (12)

Although Parker [24, 25] assumed an outbred population, Ineq. (12) applies to inbred focal individuals if relatedness between *M1* and *F1* (*r_M1,F1_*) is defined as the regression coefficient of relatedness (Eqs 3 and 4). Inequality (Eq 12) shows that the magnitude of inbreeding depression below which inbreeding with *F1* benefits *M1* decreases as *c* increases from zero. For example, if $r_{M1,F1}=\frac{1}{2}$, then inbreeding with *F1* increases *M1*’s inclusive fitness more than avoiding inbreeding with her if $\delta_{M1,F1}<\frac{2(1-c)}{3}$, or, if *M1* is inbred, then $\delta_{M1,F1}<\frac{\left(1+f_{M1}\right)\left(1-c\right)}{\left(1+f_{M1}+2f_{M1,F1}\right)}$. If *c* = 0, then the threshold is the familiar $\delta_{M1,F1}<\frac{2}{3}$ [24–26]. If *c* = 1, then *M1* cannot increase his fitness by inbreeding with *F1* [24, 26]. To our knowledge, it has not been noted that avoiding inbreeding with *F1* can increase *M1*’s inclusive fitness more than inbreeding with her even if inbreeding with *F1* increases *M1*’s likelihood of obtaining additional mating opportunities (i.e., *c* < 0). Mathematically, this occurs when $\delta_{M1,F1}>\frac{2}{3}$. It arises because the inclusive fitness benefit that *M1* receives from the outbred offspring of *F1* outweighs not only the potential cost of inbreeding in terms of *M1*’s reduced ability to sire offspring, but also some amount of inclusive fitness gain that could potentially result from access to new mates through inbreeding with *F1* (e.g., in a polygynous system).

Parker’s model that includes the opportunity cost *c* (Ineq. 12) again assumes reciprocal inbreeding avoidance by the two focal individuals *M1* and *F1*. We again relax this assumption by introducing a relative *M2* and determine the effect on *M1*’s inclusive fitness gain from inbreeding versus avoiding inbreeding with *F1*. Both *M1* and *M2* will then experience an opportunity cost *c* of breeding with *F1*; moreover, these costs are not independent if *r*_*M*1,*M*2_ > 0. Specifically, *M1* will experience an indirect inclusive fitness cost of avoiding inbreeding with *F1* if he is related to *F1*’s alternative mate *M2* (i.e., *r*_*M*1,*M*2_ > 0). In this case, *M1*’s inclusive fitness will decrease because of his relative *M2*’s reduced opportunity to sire offspring with other females. The indirect cost to *M1* equals the opportunity cost to *M2* ($\frac{nc}{2}$) multiplied by *r_M1,M2_*. Extending our model that includes *M2* (Ineq. 7) to include direct and indirect male opportunity costs shows that *M1* increases his inclusive fitness by inbreeding with *F1* instead of avoiding inbreeding with her if,

$$\begin{array}{ccc} & & \underset{\begin{array}{c} \text{Fitness}\\ \text{increment}\;\text{when}\\ \text{M1}\;\text{inbreeds} \end{array}}{\underset{︸}{\frac{n}{2}(1+r_{M1,F1})}}\underset{\begin{array}{c} \text{Reduced}\;\text{fitness}\\ \text{of}\;\text{M1}\;\&\;\text{F1}’\text{s}\\ \text{offspring} \end{array}}{\underset{︸}{\left(1-\delta_{M1,F1}\right)}}-\underset{\begin{array}{c} \text{M1}’\text{s}\\ \text{mating}\\ \text{cost} \end{array}}{\underset{︸}{\frac{nc}{2}}}>\\ & & \;\underset{\begin{array}{c} \text{Fitness}\;\text{increment}\\ \text{when}\;\text{M1}\\ \text{avoids}\;\text{inbreeding} \end{array}}{\underset{︸}{\frac{n}{2}(r_{M1,F1}+r_{M1,M2})}}\underset{\begin{array}{c} \text{Reduced}\;\text{fitness}\\ \text{of}\;\text{M2}\;\&\;\text{F1}’\text{s}\\ \text{offspring} \end{array}}{\underset{︸}{\left(1-\delta_{M2,F1}\right)}}-\underset{\begin{array}{c} \text{M1}’\text{s}\;\text{indirect}\\ \text{cost}\;\text{of}\;\text{M2}\\ \text{mating} \end{array}}{\underset{︸}{\frac{nc}{2}r_{M1,M2}}}. \end{array}$$ (13)

To explore this model, we again consider the three simple illustrative scenarios shown in Fig 3. In scenario 1, where *M1*, *F1*, and *M2* are all equally related (Fig 3A and 3B), inbreeding with *F1* increases *M1*’s inclusive fitness more than avoiding inbreeding with her if *δ*_*M*1,*F*1_ < 1 − *c*. Therefore, if there is no opportunity cost, *M1* benefits by inbreeding with *F1* when *δ*_*M*1,*F*1_ < 1, as discussed previously. But if *c* = 1, *M1* never benefits by inbreeding (consistent with Parker [24] and Waser et al. [26]). Including an opportunity cost in scenario 1 therefore decreases the inbreeding depression threshold below which *M1* benefits by inbreeding with *F1* versus inbreeding avoidance.

In scenario 2 where $r_{M1,F1}=\frac{1}{4}$, $r_{M1,M2}=\frac{1}{8}$, and *r*_*M*2,*F*1_ = 0 (Fig 3C and 3D), *δ*_*M*2,*F*1_ = 0 because *M2* and *F1* are unrelated. Rearranging Ineq. (13) reveals that *M1*’s inclusive fitness gain from inbreeding with *F1* is higher than that gained by avoiding inbreeding if $\delta_{M1,F1}<\frac{7}{10}(1-c)$. Again, inbreeding is never beneficial for *M1* if *c* = 1.

In scenario 3 where $r_{M1,F1}=\frac{1}{4}$, *r*_*M*1,*M*2_ = 0, and $r_{M2,F1}=\frac{1}{8}$ (Fig 3E and 3F), Ineq. (13) reveals that *M1* increases his inclusive fitness more by inbreeding with *F1* than by avoiding inbreeding with her if 4 > 5*δ*_*M*1,*F*1_ − *δ*_*M*2,*F*1_ + 4*c*. If *c* = 0, this condition is satisfied for *M1* if $\delta_{M1,F1}<\frac{4}{5}$ (assuming *δ*_*M*2,*F*1_ ≥ 0). But when *c* > 0, the values of *δ*_*M*1,*F*1_ for which it is satisfied become more restricted. If *c* = 1, *M1*’s inclusive fitness is never greater when inbreeding with *F1* except given unrealistically high outbreeding depression (i.e., *δ*_*M*2,*F*1_ ≫ *δ*_*M*1,*F*1_).

## Discussion

The fitness costs associated with inbreeding, primarily inbreeding depression in resulting offspring, have caused a widespread assumption among animal ecologists that inbreeding avoidance must be adaptive [10, 21, 22]. Meanwhile, empirical studies have reported a lack of inbreeding avoidance [34, 42–47], or even an apparent preference for inbreeding [48–52], causing a mismatch between expectations and data [27]. This mismatch is partially resolved by basic conceptual models of biparental inbreeding that imply that the inclusive fitness benefit of inbreeding might cause inbreeding tolerance or preference to be adaptive even given inbreeding depression in offspring fitness, and that predict sexual conflict over inbreeding [24–28].

Here we show that relaxing key restrictive assumptions, namely that focal individuals are themselves outbred, and that inbreeding avoidance is mutual, changes key quantitative and qualitative predictions of Parker’s model [24, 25]. Specifically, the zone of sexual conflict over inbreeding increased when a focal male’s inclusive fitness gain from avoiding inbreeding decreased because his rejected female relative subsequently inbred. Conversely, the zone of sexual conflict decreased when a focal male’s rejected female relative instead mated with a male that was related to the focal male but not to her, thereby increasing the focal male’s inclusive fitness through his female relative’s alternative mate choice. Consequently, the zone of sexual conflict, and thus the sexually antagonistic selection that drives coevolving mating traits [53], will depend on the distributions of inbreeding and relatedness among potential mates, and on how these potential mates interact to determine inbreeding versus inbreeding avoidance among relatives.

Puurtinen’s [28] model differs from models that assume reciprocal inbreeding avoidance [24–26]. Puurtinen [28] highlights an interesting algebraic implication of Parker’s model by deriving the stable degree of inbreeding when females mate with optimal relatives. His model assumes that optimally related mates are always available, and that sexual conflict is resolved in favour of females, with negligible male opportunity cost and linear inbreeding depression. Following from Parker’s model, Puurtinen’s [28] development also assumes that inclusive fitness effects are not conditional upon the mating decisions of relatives, so predictions might not be quantitatively applicable if this assumption is violated.

When the inclusive fitness effects of an individual’s behaviour are conditional upon the behaviour of other individuals, the effects of social interactions are non-additive [54, 55]. In such cases, the combined inclusive fitness effects of pair-wise social interactions for focal individuals cannot simply be added up. In contexts other than biparental inbreeding, the complicating effect of non-additivity has long been recognised [56], and there is growing consensus that social interactions are likely to have non-additive effects on inclusive fitness [55]. In the context of biparental inbreeding, additive inclusive fitness effects are widely assumed [24–28], but are unlikely to be biologically realistic. Our extensions of Parker’s model suggest that conceptual models that assume reciprocal inbreeding avoidance [24–26] or ubiquitous availability of optimally related kin [28] will have limited predictive ability when the foundational assumption of additive inclusive fitness effects is violated. To develop a comprehensive theory of inbreeding, it is therefore necessary to move beyond pair-wise interactions between potential mates and consider inbreeding conflict in the broader context of populations characterised by complex and non-additive interactions among realistic distributions of relatives.

Non-additive inclusive fitness effects are likely prevalent in both plant and animal populations in which biparental inbreeding occurs. Most models that focus on plants consider the fitness costs and benefits of self-fertilisation versus outcrossing, but assume that non-selfing individuals inevitably outbreed with no opportunity to cross with a non-self relative (but see [57]; [11–16]). As in animal populations, when biparental inbreeding and inbreeding depression occur in plants [58–60], inbreeding conflict is expected, though very few studies have considered how such conflict might be resolved [61].

### New directions for inbreeding theory

Our extension of Parker’s model of inbreeding between *M1* and *F1* to include another relative *M2* changed inclusive fitness outcomes both quantitatively and qualitatively, suggesting that comprehensive new theory regarding the evolution of biparental inbreeding needs to be developed to make quantitative predictions about inbreeding strategies. Our extension was minimal; to keep the model tractable while making our conceptual point, we only allowed three individuals to be related (e.g., *F2* was assumed to be unrelated to *M1*, *F1*, and *M2*; Fig 1). In natural populations, multiple potential mates of both sexes might be related to each other to different degrees. A more comprehensive theory of biparental inbreeding that incorporates realistic variation in relatedness arising from any mating system and potential feedbacks between relatedness and (inclusive) fitness is likely needed to make useful quantitative predictions. The complexity inherent in modelling multiple interacting individuals that are related to different degrees due to internally consistent ancestry means that further extensions to simple algebraic models will quickly become intractable. Below we show how our model of inbreeding among more than two relatives creates novel predictions regarding inbreeding conflict among nuclear family members, and outline key steps towards predictive evolutionary models of inbreeding strategies.

#### Family dynamics

The dynamics of interactions among nuclear family members, including mating strategies, are of major interest in evolutionary and behavioural ecology [62–65]. Relaxing the assumption of additive inclusive fitness effects may alter predictions regarding within-family sexual conflict over inbreeding. For example, Parker’s model predicts the thresholds of inbreeding depression below which mother-son inbreeding increases inclusive fitness more than inbreeding avoidance to be $\delta_{M1,F1}<\frac{1}{3}$ and $\delta_{M1,F1}<\frac{2}{3}$ for an outbred mother and son, respectively (Ineqs. 1,2), implying mother-son conflict over inbreeding. But if following inbreeding avoidance by her son, the mother mates with her son’s father (as is likely in many socially monogamous animal mating systems) rather than outbreeding with a mutually unrelated male, her son’s inclusive fitness will increase because of his relatedness to his father [66]. If the son’s mother and father are unrelated, they will produce outbred full siblings of the focal son. Inequality (Eq 7) shows that the son’s threshold for inbreeding will then be $\delta_{M1,F1}<\frac{1}{3}$ instead of $\delta_{M1,F1}<\frac{2}{3}$. The magnitude of inbreeding depression below which a son increases his inclusive fitness by inbreeding with his mother is therefore identical to that for his mother, eliminating mother-son conflict.

In contrast, father-daughter conflict over inbreeding is not predicted to be eliminated. This is because any alternative mate of a daughter that is related to her father must also be related to her. Therefore, daughters cannot outbreed with their father’s relatives in the same way that mothers can with their son’s relatives. Thus, while Waser et al. [26] suggest that mother-son inbreeding should be rarer than father-daughter inbreeding because sexual conflict is more likely to be resolved in favour of older individuals, such age effects need not be invoked if a son’s mother will likely continue to breed with his father. If a son has high confidence in the identity of both parents, he might benefit from ensuring that his parents continue to breed together to ensure the production of full siblings (i.e., “mother guarding”; [67]). If the alternative mate of a son’s mother is not his father, the son will be less closely related to his mother’s offspring, decreasing his inclusive fitness gain from avoiding inbreeding with his mother and therefore potentially leading to inbreeding conflict. This simple example illustrates that accounting for the alternative mates of relatives will be necessary for any comprehensive theory of family dynamics.

#### Male-male conflict over inbreeding with a mutual female relative

While inter-sexual conflict over inbreeding is often emphasised, the evolution of inbreeding strategy might also be affected by interactions between same-sex relatives. Our model assumed that the inbreeding strategy of any one focal male was not directly affected by the strategies of other males. Specifically, we assumed that when a focal male *M1* avoided inbreeding, the other male *M2* mated with the focal female *F1*. We further assumed that when *M1* inbred with *F1*, *M2* did not interfere. But in reality, *M1* and *M2* might mutually compete to inbreed with *F1*, or might mutually avoid inbreeding. If inbreeding conflict is generally resolved in favour of females, meaning that females are successful at avoiding inbreeding when it benefits them to do so, then there might be little selection for males to attempt inbreeding when there is sexual conflict. Alternatively, if inbreeding conflict is generally resolved in favour of males, then a focal male’s reproductive success will be affected by both his own inbreeding or inbreeding avoidance strategies and the strategies of his male relatives. For example, competition between *M1* and *M2* may affect the inclusive fitness benefits of inbreeding. Details of the mating system are therefore likely to strongly influence the resolution of sexual conflict [25, 68].

As an illustrative example, if *M1* and *M2* are equally related to *F1* and inbreeding depression is strong, then *M1* and *M2* will both benefit from mutual inbreeding avoidance. Both males will lose fitness if either inbreeds with *F1*, but each will lose more if he avoids inbreeding with *F1* but his male relative inbreeds with *F1*. The decision for each male to inbreed with *F1* or not can be modelled using a game-theoretic framework where payoffs are proportional to the inclusive fitness benefits for different mating situations. A full game-theoretic model is beyond the scope of this paper, but a simple example illustrates a basic framework.

For simplicity, we assume an outbred population and define *r* = *r*_*M*1,*F*1_ = *r*_*M*2,*F*1_ = *r*_*M*1,*M*2_ and *δ* = *δ*_*M*1,*F*1_ = *δ*_*M*2,*F*1_. To further simplify the notation, we assume *F1* produces two offspring (*n* = 2), so $\frac{n}{2}=1$ when calculating inclusive fitness; changing this assumption does not affect the relative payoffs of different mating situations and therefore does not affect the generality of the model. We further assume no opportunity cost of inbreeding (i.e., *c* = 0). If both *M1* and *M2* avoid inbreeding, we assume *F1* outbreeds with a different unrelated male. Both *M1* and *M2* therefore have an indirect fitness benefit of *r* ($\frac{nr}{2}$). If *M1* inbreeds but *M2* does not, then *M1*’s inclusive fitness is (1 + *r*)(1 − *δ*), while *M2*’s inclusive fitness is 2*r*(1 − *δ*), with the opposite inclusive fitness payoffs if *M1* avoids inbreeding and *M2* inbreeds. If both *M1* and *M2* attempt to inbreed, we assume no shared paternity and that each has an equal chance of success; therefore each receives an expected inclusive fitness payoff of $\frac{1}{2}\left[(1+r)(1-\delta )+2r(1-\delta )\right]=\frac{1}{2}(1+3r)(1-\delta )$.

These payoffs can be placed in a matrix (Table 1) to determine the evolutionary stable strategy (ESS [69]) of inbreeding for *M1* and *M2* in terms of *r* and *δ*. For many combinations of *r* and *δ*, the ESS for each male is pure (i.e., always inbreed or avoid inbreeding). For example, if *M1*, *F1*, and *M2* are full siblings of outbred parents ($r=\frac{1}{2}$), and inbreeding depression is complete (*δ* = 1), then *M1* benefits only if both he and *M2* avoid inbreeding. Inbreeding avoidance is therefore an ESS for *M1* (i.e., *M1* should always avoid inbreeding), but the maximum payoff is only realised if *M2* also avoids inbreeding. In contrast, if there is no inbreeding depression (*δ* = 0), attempting to inbreed always gives *M1* a higher payoff, even if *M2* also attempts to inbreed.

**Table 1:** A general payoff matrix for male relatives (*M1*, *M2*) for either inbreeding or avoiding inbreeding with a female (*F1*) of equally close relatedness (*r*).

**M2 strategy**	**M1 strategy**	
	**Avoid**	**Inbreed**
**Avoid**	*r*, *r*	2*r*(1 − *δ*), (1 + *r*)(1 − *δ*)
**Inbreed**	(1 + *r*)(1 − *δ*), 2*r*(1 − *δ*)	$\frac{1}{2}(1+3r)(1-\delta )$, $\frac{1}{2}(1+3r)(1-\delta )$

Unless both *M1* and *M2* avoid inbreeding, inbreeding depression (*δ*) will reduce the fitness of *F1*’s offspring. If both males avoid inbreeding, for simplicity, *F1* is assumed to mate with an unrelated male. *F1* is assumed to produce 2 offspring.

Interestingly, a mixed strategy, in which inbreeding avoidance is probabilistic, is an ESS for a narrow range of intermediate values. For example, if $\delta =\frac{13}{20}$, then both *M1* and *M2* have an inclusive fitness benefit of $\frac{1}{2}$ in the case of mutual inbreeding avoidance, but only $\frac{5}{16}$ if both attempt to inbreed. If *M1* inbreeds but *M2* avoids inbreeding, then *M1* will receive a payoff of $\frac{21}{40}$, greater than if both mutually inbreed or avoid inbreeding. In contrast, *M2* receives a payoff of only $\frac{7}{20}$. This payoff matrix has the format of a Hawk-Dove game [69, 70] in which the ESS for *M1* (and *M2*) is mixed (i.e., for each interaction, a strategy is selected with some probability). Instead of an absolute inbreeding avoidance, preference, or tolerance strategy, a probabilistic strategy might therefore be predicted in some circumstances. This basic model makes multiple restrictive assumptions, but it suggests that a game-theoretic approach might be useful for understanding mating conflicts among male relatives.

### Predictive models of inbreeding strategy

Our models imply that the inclusive fitness costs and benefits of inbreeding versus avoiding inbreeding will vary among individuals depending on their interactions with multiple different relatives of both sexes, and on the degree to which focal individuals are themselves inbred. Understanding these costs and benefits and their combined consequences for the evolution of inbreeding strategies therefore requires consideration of not only the relatedness of an individual to its potential mate(s), but also the relatedness between the individual and the subsequent mates of rejected relatives. Knowledge of the distribution of relatedness within a population is therefore likely to be critical for understanding the evolution of inbreeding strategies. This distribution will in turn depend on the distribution of relatedness in previous generations and on previously realised inbreeding strategies and inbreeding loads, thereby generating complex feedbacks between inbreeding strategy, load, and relatedness. Because of the complexity inherent in these systems, neither our conceptual model presented here nor its predecessors [24–28] should be interpreted as providing quantitatively accurate predictions of inbreeding depression thresholds underlying inbreeding avoidance versus preference that might apply to empirical systems (as is sometimes attempted [31–34]).

A comprehensive theory of biparental inbreeding strategy will require new models that explicitly consider interactions among numerous relatives [10]. Because sexual conflict over inbreeding is predicted [10, 24], the ultimate evolution of inbreeding strategy will depend on the degree to which conflict is resolved in favour of males versus females [25]. Inbreeding strategy might also depend on the encounter rate between potential mates. Like most predecessors [24–26, 28], our model implicitly assumes that individuals have a rapid encounter rate among potential mates. Kokko and Ots [27] relaxed this assumption, and showed that greater inbreeding depression is required to make inbreeding avoidance beneficial given sequential rather than simultaneous mate encounters (see also [29]). A general, comprehensive theory of inbreeding must therefore propose some resolution of sexual conflict and consider appropriate aspects of life-history and population ecology.

Sexual conflict and interactions among multiple non-self relatives are particular to biparental reproduction rather than self-fertilisation, but both types of inbreeding increase the expression of inbreeding load causing inbreeding depression in offspring [1, 2, 7, 12]. Inbreeding depression may decrease inbreeding load by exposing deleterious homozygous recessive alleles to selection [9, 71]. Resulting purging of deleterious recessive alleles may in turn affect the inclusive fitness benefit of inbreeding versus avoiding inbreeding causing inbreeding strategy and inbreeding load to coevolve [12, 72, 73]. The consequences of this coevolution have been modelled extensively with respect to outcrossing versus selfing [12, 18, 72, 74–76], but have not yet been modelled for the evolution of biparental inbreeding strategies [10]. Future theoretical developments will therefore need to explicitly consider coevolution between biparental inbreeding strategy and inbreeding load.

Predictive models of biparental inbreeding evolution cannot be simple, but their complexity need not preclude generality [77]. Tractable approaches for developing inbreeding theory might include game-theoretic models, or individual-based models that explicitly track ancestry and inbreeding load, and thereby incorporate feedbacks among relatedness, load, and inbreeding strategy.

## Supporting Information

S1 File Consequences of assuming that inbreeding depression in offspring is a linear function of parental kinship. (PDF)
