# Supplementary material for: What Happens after Inbreeding Avoidance? Inbreeding by Rejected Relatives and the Inclusive Fitness Benefit of Inbreeding Avoidance
Source: PLoS One. 2015 Apr 24;10(4):e0125140. doi: 10.1371/journal.pone.0125140 (PMC4409402; doi:10.1371/journal.pone.0125140)
Supplement: S1 File — (PDF) [file pone.0125140.s001.pdf]

# What happens after inbreeding avoidance? Inbreeding by rejected relatives and the inclusive fitness benefit of inbreeding avoidance.

## Supplemental Material: Consequences of assuming that inbreeding depression in offspring is a linear function of parental kinship

A. Bradley Duthie[1*], Jane M. Reid[2],

**1 Institute of Biological and Environmental Sciences, School of Biological Sciences, Zoology Building, Tillydrone Avenue, University of Aberdeen, Aberdeen AB24 2TZ, United Kingdom**

**2 Institute of Biological and Environmental Sciences, School of Biological Sciences, Zoology Building, Tillydrone Avenue, University of Aberdeen, Aberdeen AB24 2TZ, United Kingdom**

∗ **E-mail: aduthie@abdn.ac.uk**

# Abstract

In the main text, we assume a log-linear relationship between parental kinship and inbreeding depression in offspring. This supplemental material provides an overview of inbreeding depression thresholds for a focal male *M1* and female *F1* when inbreeding depression is a linear function of parental kinship. We show that the qualitative conclusions obtained based on log-linear functions do not differ when a linear function is assumed instead. Our qualitative conclusions are therefore robust to different relationships between parental kinship and inbreeding depression in offspring.

# Inbreeding depression as a linear function of inbreeding

Although offspring fitness ($W$) is typically assumed to decrease log-linearly with offspring coefficient of inbreeding, it can also be modelled as a linear function such that for an offspring produced by $M1$ and $F1$, $W = -\beta_0 - \beta_1 f_{M1,F1}$ [1, 2].

The fitness of inbred offspring relative to outbred offspring produced by $M1$ and $F1$ can then be defined by,

$$\beta_0 - \beta_1 f_{M1,F1} = (1 - \delta_{M1,F1}). \tag{S1-1}$$

This linear model fits empirical data comparably well to the log-linear model for inbreeding magnitudes typical of animal populations with biparental sexual reproduction [1, 3].

In the case of the focal $M1$ and female $F1$, and $F1$'s alternative male $M2$, inbreeding with $F1$ increases $M1$'s inclusive fitness more than avoiding inbreeding under the following condition,

$$\frac{n}{2}\left(1 + \frac{2f_{M1,F1}}{1+f_{M1}}\right)(-\beta_0 - \beta_1 f_{F1,M1}) > \frac{n}{2}\left(\frac{2\left(f_{M1,F1} + f_{M1,M2}\right)}{1+f_{M1}}\right)(-\beta_0 - \beta_1 f_{M2,F1}). \tag{S1-2}$$

The variable $\beta_1$ can be isolated to find the threshold below which inbreeding with $F1$ increases $M1$'s inclusive fitness more than avoiding inbreeding,

$$\beta_1^{M1} < \frac{-\beta_0\left(1 + f_{M1} - 2f_{M1,M2}\right)}{f_{M1,F1}\left(2f_{M2,F1} - 1 - f_{M1}\right) + 2\left(f_{M2,F1}f_{M1,M2} - f_{M1,F1}^2\right)}. \tag{S1-3}$$

Inequality (S1-3) is equivalent to the log-linear inequality (10). Assuming that $\beta_0 = 1$, inequality (S1-3) can be used to determine the inbreeding depression slope ($\beta_1$) below which a focal male $M1$ will benefit

by inbreeding with $F1$ assuming that she would otherwise breed with an alternative male $M2$ who might be related to $F1$, $M1$, or both.

The linear slope of inbreeding depression above which $F1$'s inclusive fitness is greater when inbreeding with $M1$ rather than $M2$ ($\beta_1^{F1}$) can be similarly found. In this case, $F1$ will increases her inclusive fitness by inbreeding with $M1$ instead of avoiding inbreeding with $M1$ and breeding with $M2$ instead if,

$$\frac{n}{2}\left(1+\frac{2f_{F1,M1}}{1+f_{F1}}\right)\left(-\beta_0-\beta_1 f_{F1,M1}\right) > \frac{n}{2}\left(1+\frac{2f_{F1,M2}}{1+f_{F1}}\right)\left(-\beta_0-\beta_1 f_{F1,M2}\right). \tag{S1-4}$$

Again, $\beta_1$ can be isolated to find the conditions under which breeding with $M1$ increases $F1$'s inclusive fitness more than breeding with $M2$,

$$\beta_1^{F1} < \frac{2\beta_0}{1+2\left(f_{F1,M1}+f_{F1,M2}\right)+f_{F1}}. \tag{S1-5}$$

Again assuming that $\beta_0 = 1$, inequality (S1-5) can be used to determine the slope of inbreeding depression ($\beta_1$) below which a focal female $F1$ will benefit by inbreeding with $M1$ versus $M2$. Figure S1-1 is equivalent to Figure 4 in the main text, showing that key qualitative results do not change if linear, rather than log-linear, inbreeding depression is assumed.

# References


1. Ralls K, Ballou JD, Templeton A (1988) Estimates of lethal equivalents and the cost of inbreeding in mammals. Conservation Biology 2: 185–193.

2. Puurtinen M (2011) Mate choice for optimal (k)inbreeding. Evolution 65: 1501–1505.

3. Makov E, Bittles AH (1986) On the choice of mathematical models for the estimation of lethal gene equivalents in man. Heredity 57: 377–380.

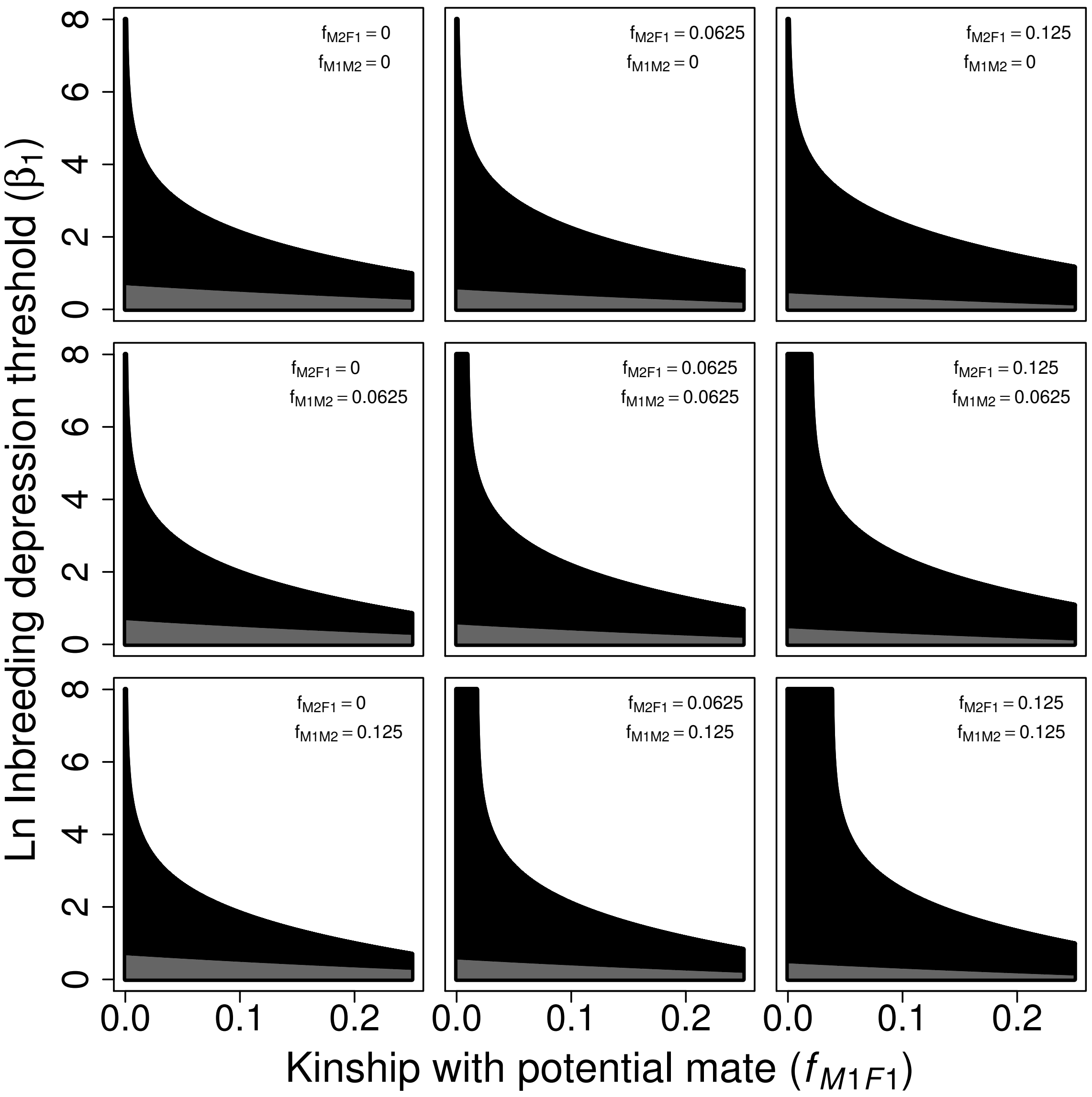


**Figure 1.** Zones of parameter space in which inbreeding versus inbreeding avoidance is predicted to increase male and female inclusive fitness given varying kinship between $M1$, $F1$, and $M2$. Inbreeding depression thresholds on the y-axis are shown on a natural log scale for clarity. These thresholds illustrate the values below which $M1$ and $F1$ have a higher inclusive fitness by inbreeding instead of avoiding inbreeding. If $M1$ and $F1$ do not inbreed, $F1$ is assumed to breed with $M2$, who may or not be related to $M1$ or $F1$. The kinship between $M1$ and $F1$ ($f_{M1,F1}$) increases along the x-axis of all plots. $f_{M2,F1}$ and $f_{M1,M2}$ increase through 0, 0.0625, and 0.125 across left to right columns and top to bottom rows, respectively. Areas where neither sex, both sexes, and males only benefit from inbreeding are shown in white, grey, and black, respectively. Black regions left of the vertical asymptote exist for conceptual clarity and correspond to regions in which outbreeding depression is required for inbreeding avoidance to be beneficial. For the purpose of illustration, focal individuals are assumed to be outbred.
